# Governance in mental healthcare policies during the COVID-19 pandemic in Mexico

**DOI:** 10.3389/fpubh.2023.1017483

**Published:** 2023-03-06

**Authors:** Lina Diaz-Castro, Jose Carlos Suarez-Herrera, Oscar Omar Gonzalez-Ruiz, Emanuel Orozco-Nunez, Mario Salvador Sanchez-Dominguez

**Affiliations:** ^1^National Institute of Psychiatry Ramon de la Fuente Muñiz, Directorate of Epidemiological and Psychosocial Research, Mexico City, Mexico; ^2^KEDGE Business School, Entrepreneurship and Sustainable Development, Department of Strategy, Marseille, France; ^3^National Institute of Public Health, Health Systems Research Center, Cuernavaca, Mexico; ^4^Medical Sciences, National Institute of Public Health, Health Systems Research Center, Cuernavaca, Mexico

**Keywords:** governance, policy-makers, mental disorders, decision-making, public policy

## Abstract

The COVID-19 pandemic has become the greatest burden of disease worldwide and in Mexico, affecting more vulnerable groups in society, such as people with mental disorders (MD). This research aims to analyze the governance processes in the formulation of healthcare policies for people with MD in the face of the COVID-19 pandemic. An analytical qualitative study, based on semi-structured interviews with key informants in the healthcare system was conducted in 2020. The study followed the theoretical-methodological principles of the Governance Analytical Framework (GAF). The software ATLAS.ti-V.9 was used for inductive thematic analysis, classifying themes and their categories. To ensure the proper interpretation of the data, a process of triangulation among the researchers was carried out. The findings revealed that in Mexico, the federal Secretary of Health issued guidelines for mental healthcare, but there is no defined national policy. Decision-making involved multiple actors, with different strategies and scopes, depending on the type of key-actor and their level of influence. Majority of informants described a problem of implementation in which infection control policies in the psychiatric population were the same as in the general populations which decreased the percentage of access to healthcare during the pandemic, without specific measures to address this vulnerable population. The results suggest that there is a lack of specific policies and measures to address the needs of people with mental disorders during the COVID-19 pandemic in Mexico. It also highlights the importance of considering the role of different actors and their level of influence in the decision-making process.

## 1. Introduction

Currently, the pandemic due to the new coronavirus COVID-19 is the cause of the greatest burden of the disease worldwide as well as in Mexico (1). Due to the characteristics of its spread and the health measures for its control, it can increase the vulnerability of people with mental disorders (MD). Different measures of social isolation can affect the mood of these people with the consequent aggravation of their different psychopathological conditions. Consequently, the families and institutions that are protected by these people must offer specific assistance and monitoring to each of them (2). Governments in all countries have formulated various policies in health systems to address the Public Health Emergency of International Concern (PHEIC) due to COVID-19, but responsiveness has represented a global challenge (3). This situation highlighted the lack of cohesion that exists between the institutions of the Mexican National Health System (NHS). The Mexican NHS is composed and financed by both the public and private sectors. The public sector provides care to (1) people affiliated with social security (who receive a formal salary) through the Mexican Institute of Social Security (IMSS—from now on acronyms are in Spanish), the Institute of Security and Social Services of State Workers (ISSSTE), the Armed Forces (SEDENA and SEMAR) and the Mexican Petroleum (PEMEX). This represents 48.3 million people funded by employers, workers and the federal government; and (2) people without social security (who do not have a formal salary) who receive care from the Secretary of Health, Federal (SSA) or States' (SESA), and which are the object of this study. Up until the year 2019, the healthcare of these 58 million people had been financed in two ways, (1) by the federal government and the state governments through the System of Social Protection of Health and its program “Popular Insurance” (“Seguro Popular”), and/or (2) the out-of-pocket expenses of the user at the point of service (4).

At that time, when the COVID-19 pandemic was declared (March 2020), the NHS was implementing a new scheme for the provision of health services to the population without social security. This has implied stagnation in the programmed implementation of the reform strategies for the period 2019–2024, and instead, the mitigation of the pandemic was established as a priority programmatic axis (5). As a central strategy of the response, a process called “hospital reconversion” was carried out, which prioritized COVID-19 care first, without defining or informing users of procedures for monitoring the routine demand for medical services in general.

In addition, half of the people who receive medical care due to some MD, especially severe, do so in psychiatric hospitals, which suffer from low budget and resources to provide quality care (6, 7), furthermore, in a pandemic context people with mental pathology have a greater probability of getting sick with another chronic pathology than the general population (8–10). In consequence, the Mexican NHS has two challenges to guaranteeing care in psychiatric hospitals. On one hand, there are long-stay psychiatric hospitals with a confined population. On the other hand, psychiatric hospitals with functioning like that of a general hospital. First, patients are more vulnerable to being infected; in such a situation, measures should be taken to prevent contagion in a gated community (11), in the latter, they must also ensure the continuity of psychiatric care that allows treatment adherence, especially for serious conditions, monitoring the risk of aggressiveness toward oneself or others, as well as detecting symptoms associated with living in quarantine such as stress, anxiety or depression due to the current pandemic (12).

Several studies have reported strategies to ensure the medical care of the mentally ill during the epidemic with measures such as reducing the length of stay, reducing visits to admitted patients, reducing outpatient care and in hospitalized patients, timely detection of high-risk or suspected COVID-19 patients and isolation of positive patients (12–14). In Mexico, the strategy of offering psychosocial support was aimed at the general population that does not have COVID-19, people with COVID-19 who are isolated at home and/or in hospital, the population that referred COVID-19, relatives and caregivers of patients with COVID-19, health personnel and lifeguards before the emergency; it included psychological first aid and crisis intervention, as well as emotional support. This strategy considers it essential to try to have a telephone number for psychological or psychiatric emergencies and to provide care to mental health personnel (15). But it is unknown what the scope of this national strategy has been within the country, how decision makers adopted it or formulated new policies for the protection and care of people with MD on the understanding that comprehensive mental health policies must be implemented to respond to the daily healthcare needs of the people with MD, while still responding in the same way, to health emergencies, such as the current pandemic (7, 16, 17).

One way to address and support policy decision-making is through strengthening health system governance (18–20). Globally, governance in healthcare refers to the implementation of policies and practices that promote equitable health systems (21, 22). Other international organizations equate the concept of governance with stewardship, or co-management, to refer to concerted actions that promote and protect public health (23), or with an intersectoral governance approach, that refers to the coordination of multiple sectors to address health problems (24, 25). These definitions have a normative approach. In this research an approach to governance as an intermediate analytical variable is proposed, a generalizable concept, which refers to the process of agreement in decision-making, in which all the actors of the health system, suppliers and consumers intervene, with well-defined roles, to meet the demands of mental healthcare, with the focus of patient-centered care based on evidence, responsibility and accountability (25, 26). Incorporating this governance approach poses challenges for health systems in their communities, providing essential services both in the short term (after a disaster or pandemic, for example, the COVID-19) and in the long term in terms of public health. The role and critical nature of healthcare facilities means that they have significant impacts on communities, and the decisions affect the natural system in which we all live and have an impact on the future environmental. These impacts do not affect communities in the same way. Vulnerable populations such as people with MD suffer the effects on the environment due to factors such as access to resources and social determinants of health that influence health risks and outcomes. Populations with MD are less able to deal with the consequences for human health. In this sense, the approach of the Governance Analytical Framework (GAF) in the field of public health, visualizes governance as a social fact, endowed with analyzable and interpretable characteristics: the problem from a governance approach, the actors, social norms, the process, and the nodal points (27). Therefore, it will be the approach that we used.

The objective of this research is to analyze the governance process implemented in the formulation of policies for healthcare of people with mental disorders in the face of the COVID-19 pandemic.

## 2. Materials and methods

**Figure 1:**
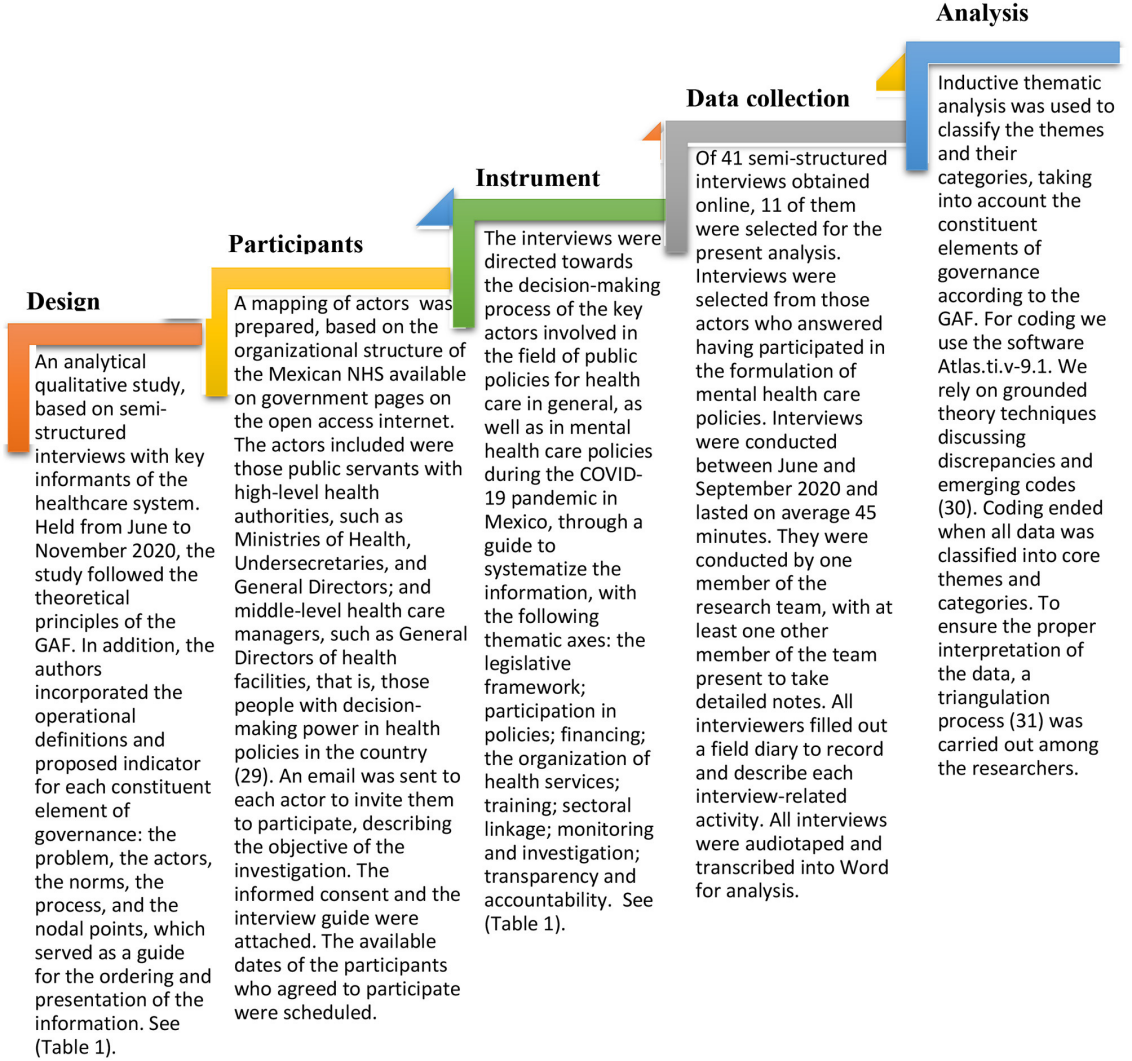
Applied research methodology.

A qualitative research methodology used since the 1960s, is proposed. It is a systemic and essentially critical methodology in all its phases, from its data collection instruments to the quality criteria, such as classic validity and reliability. Given the intricate web of variables (antecedents, intervening and interacting), a critical analysis is essential throughout the research process (28). By applying this methodology, precise information is obtained on how the different social actors perceive, interact and make decisions in the formulation of policies for the care of people with MD, according to the thematic categories of analysis proposed in the GAF: the problem, the actors, the social norms, the process, and the nodal point, (see Table 1), with the method described in the Figure 1.

**Table 1:** Governance Analytical Framework (GAF).

**Governance element**	**Categorical definition**	**Categorical dimension**	**Analytical properties or subcategories**	**Qualitative indicators**
1. Governance problem	Phenomenon under analysis	Health problem	Characterization and/or scope of health outcomes	Tracer(s)
2. Key actor: Actors of the healthcare system	Every individual involved in the institutional network of mental healthcare, with (or without) resources of power	Type of actor	Strategic Actor	Yes/No
			Stakeholder	Yes/No
		Academic and managerial background	Profession/Academic Degree	Undergraduate/Postgraduate
			Managerial Type	Technical/Human/Conceptual
			Managerial Level	High, Low
		Leadership skills	Management	High, Middle, Low
			Administrative	High, Middle, Low
			Governance	High, Middle, Low
		Status	Formal	Yes/No
			Informal	Yes/No
		Positioning	Facilitator/Opponent	Yes/No (Unknown)
3. Process (decision making). Interview Guide: Level of involvement of the key actor and power in the formulation of public policies in mental health	Power Resources: Ability/capacity to push, impede or disrupt the functioning of rules or procedures in the formulation and implementation of mental healthcare policies and programmes	Power resources in Mental Health Policy	Symbolic Resources	Yes/No
			Monetary Resources	Yes/No
			Social Capital Resources	Yes/No
		Level of Power (Nature of the transaction)	Negotiation	Yes/No; High/Middle/Low
			Direction or Management	Yes/No; High/Middle/Low
			Distribution or Sharing	Yes/No; High/Middle/Low
			Reciprocity	Yes/No; High/Middle/Low
		Application of power level (in practice)	Knowledge of the legal framework and capacity to modify it	Yes/No; High/Middle/Low
			Level of involvement in the formulation of mental healthcare policies	Yes/No; High/Middle/Low
			Ability to obtain and decide on the use and allocation of resources	Yes/No; High/Middle/Low
			Capacity to monitor strategies -development and outcomes -implementation of policies	Yes/No; High/Middle/Low
			Level of participation in human resources training and capacity building needs.	Yes/No; High/Middle/Low
			Capacity to convene governmental and non-governmental organizations in society	Yes/No; High/Middle/Low
			Capacity to generate and disseminate information on mental health	Yes/No; High/Middle/Low
			Involvement in the mechanism of transparency and accountability	Yes/No; High/Middle/Low
4. Nodal points: Spaces and rules where actors interact and how they are applied in practice	Spaces (or interfaces) where several processes, stakeholders and norms converge, producing different effects on a studied problem	Level of agreement in the interaction process	Formal Norms: constitutive/regulatory	Yes/No
			Actors' Behavior	Proactive/Passive
			Modifications by collective action	Interaction/Transaction
				Conflictive/Collaborative
		Scenario of interaction	Negotiation spaces	Physical/Virtual
			Effects	Isolated/Interactive
			Scope	Local/State/National
5. Social norms: Rules that influence decision-making processes	Successions through which the interrelationship between stakeholders, norms and nodal points pass	Social Norms (game rules or decisions)	Standards: Formal, Informal	Yes/No
			Legally recognized	Dependent/Independent
			Stakeholder practice	Acknowledged Yes/No
			Stakeholder authority	Yes/No

A theoretical-methodological approach to its constituent elements.

Own elaboration.

## 3. Societal benefits of the research

The results of this article allow us to observe, beyond its initial objective, some key aspects that may be of marked interest for the future responses to certain societal challenges in the field of Governance and Global Mental Health:

First, we have realized the importance of developing a comprehensive holistic model (although adaptable to the characteristics of each territory) of Global Health, encompassing a global vision of planetary challenges (32). This requires the integration of interactions between multiple actors, from both bottom-up and top-down perspectives, anchored in an integrative governance framework and supported by an interdisciplinary and intersectoral approach (33, 34).

Secondly, through this article we realize that new, more inclusive (35) and reflexive (36) governance models are necessary to face the complexity of contemporary Global Health challenges. On the one hand, inequalities affect the health and wellbeing of populations at global, regional, and national levels. An inclusive approach to governance in Global Health is a potential way to include all key actors and thus reduce inequalities (33, 35).

Finally, in a multi-actor and multi-scale environment, it is imperative to establish the foundations of a methodological framework in empirical bioethics that can serve as a starting point for building a reflective governance model in the field of Global Health. This process of ongoing critical thinking involves “mapping, framing and shaping” the dynamics of interests and perspectives that could jeopardize a collaborative scenario (36). Finally, the conclusion of this article clearly shows us the need to develop governance models in the field of Global Health with clearly defined social purposes, allowing key actors to collectively build sustainable decision-making processes, more adapted to the needs of populations and our planet.

## 4. Results

### 4.1. The problem from a governance approach

In Mexico, the SSA, as the sole governing body of the NHS, issued guidelines and recommendations for mental healthcare during the COVID-19 pandemic. They recommended providing continuous healthcare for the mentally ill, but no defined national policy or specific actions for such care were issued. The mental healthcare scenario included multiple actors with different strategies throughout the country, and of different scopes, depending on both the type of key actor and the characteristics that accompany their decision-making, as shown in the analysis of the interviews, according to the analytical categories.

### 4.2. The key actors of the Mexican NHS

Eleven key actors of the NHS, three participants from the federal level (27%) and eight from the state level (73%), according to the other geographical regions of the country (37). The stakeholder mapping included six actors from the SESA, three actors from the ISSSTE and two actors from the SSA.

According to his position in the Mexican NHS, four actors were Ministries of Health, one Undersecretary, two General Directors, two Medical Directors, and two Medical Subdelegates. The state and federal high-level health authorities are those who participate in the policymaking. The participation of local or municipal actors was not found. Table 2 shows the characteristics of the participants.

**Table 2:** Characterization of the key actors in the Mexican healthcare system.

**Category of analysis**	**Key actors in mental healthcare policy**										
	**NOE1ss**	**FE2ss**	**CS1ss**	**OE1ss**	**CN1ss**	**OE3ss**	**FE1ss**	**NOE8ss**	**NOE2is**	**SE1is**	**FE6is**
**Type of actor**											
Strategic Actor	+	+	+	+	+						
Stakeholder						+	+	+	+	+	+
**Academic and managerial background**											
Profession degree											
Academic degree	+	+	+	+	+	+	+	+	+	+	+
**Leadership skills**											
Management	+++	+++	+++	+++	+++	+++	+	+	++	++	++
Administrative	+++	+++	+++	+++	+++	+++	+	+	++	++	++
Governance	+	+++	+++	++	++	+	+	+	+	+	+
**Status**											
Formal	+	+	+	+	+	+	+	+	+	+	+
Informal											
**Positioning**											
Facilitator	+	+	+	+	+	+	+	+	+	+	+
Opponent											

NOE1ss, Actor 1, Northwest region, Secretary of Health; FE2ss, Actor 2, Federal, Secretary of Health; CS1ss, Actor 1, South Central region, Secretary of Health; OE1ss, Actor 1, Western region, Secretary of Health; CN1ss, Actor 1, North Central region, Secretary of Health; OE3ss, Actor 3, Western region of Mexico, Secretary of Health; FE1ss, Actor 1, Federal, Secretary of Health; NOE8ss, Actor 8, Northwest region, Secretary of Health; NOE2is, Actor 1, Northwest region, Institute of Security and Social Services of State Workers; SE1is, Actor 1, Southeast region, Institute of Security and Social Services of State Workers; FE6is, Actor 6, Federal, Institute of Security and Social Services of State Workers.

+ Feature presence.

+ Low level, ++ Middle level, +++ High level.

The actors recognized having leadership in the formulation of main policies. The Ministries and Undersecretaries acknowledged leadership in decision-making and in managerial skills, concerning the direction of policies in their field of competence. Directors and Medical Subdelegates, administrative skills for the development and implementation of policies were identified (see Table 3). Although all actors positioned themselves as facilitators of federal policies on mental health, it was discerned that two actors remained passive, regardless of decisions.

**Table 3:** Social norms and scope of decision-making by key actors in Mexico's health system.

**Category of analysis**	**Key actors in mental healthcare policy**										
	**NOE1ss**	**FE2ss**	**CS1ss**	**OE1ss**	**CN1ss**	**OE3ss**	**FE1ss**	**NOE8ss**	**NOE2is**	**SE1is**	**FE6is**
**Process (decision-making)**											
**Power resources**											
Symbolic	+	+				+	+	+	+	+	+
Monetary	+			+	+	+			+		+
Social capital	+	+	+	+	+						
**Level of power**											
Negotiation											
Direction	++	+++	+++	+	++	+		+			+
Distribution				+					+	+	+
Reciprocity							+				
**Application of power**											
Knowledge of the legal framework	+++	+++	+++	+++	+++	++	+	+	+	++	++
Capacity to modify the legal framework	+++	++	+++	+++	+++	+	+	+	+	+	+
Level of involvement in the policy formulation	++	+++	+++	+	++	+	+	+	+	+	+
Allocating resources for policies	+++	+	+++	++	++	+	+	+	+	+	+
Monitoring development and implementation policy	++	+	++	++	++	++	++	+	++	++	++
Human resources training/capacity building	+++	+	+++	+	+++	++	++	+	+++	++	+++
Capacity to convene organizations	++	+++	++	+	++	++	+	+	+	+	++
Generate and disseminate information	++	+++	++	++	++	++	+	+	+	+	+
Apply mechanisms of transparency	+++	+++	+++	+++	+++	+++	++	++	++	++	++
Apply mechanisms of accountability	++	+++	++	++	++	+	+	+	+	+	+
**Nodal points**											
**Formal norms**											
Constitutive	*	*	*	*	*	*		*	*		*
Regulatory	*	*	*	*	*	*	*	*	*	*	*
**Actors' behavior**											
Proactive	*	*	*	*	*	*	*				
Passive									*	*	
**Category of analysis**	**NOE1ss**	**FE2ss**	**CS1ss**	**OE1ss**	**CN1ss**	**OE3ss**	**FE1ss**	**NOE8ss**	**NOE2is**	**SE1is**	**FE6is**
**Modification by collective action**											
Interaction		*		*	*	*	*	*	*	*	*
Conflictive											
Collaborative	*			*				*		*	
Transaction	*		*								
**Negotiation spaces**											
Physical		*					*	*			
Virtual	*	*	*	*	*	*	*	*	*	*	*
**Effects**											
Isolated						*	*	*	*	*	
Interactive	*	*	*	*	*		*				*
**Scope**											
Local						*	*	*	*	*	
State	*		*	*	*						*
Federal		*									
**Social norms**											
Formal	*	*	*	*	*	*	*	*	*	*	*
Informal	*					*	*	*		*	

NOE1ss, Actor 1, Northwest region, Secretary of Health; FE2ss, Actor 2, Federal, Secretary of Health; CS1ss, Actor 1, South Central region, Secretary of Health; OE1ss, Actor 1, Western region, Secretary of Health; CN1ss, Actor 1, North Central region, Secretary of Health; OE3ss, Actor 3, Western region of Mexico, Secretary of Health; FE1ss, Actor 1, Federal, Secretary of Health; NOE8ss, Actor 8, Northwest region, Secretary of Health; NOE2is, Actor 1, Northwest region, Institute of Security and Social Services of State Workers; SE1is, Actor 1, Southeast region, Institute of Security and Social Services of State Workers; FE6is, Actor 6, Federal, Institute of Security and Social Services of State Workers.

^*^Feature presence. + Low level, ++ Middle level, +++ High level.

### 4.3. Decision-making process, social norms, and nodal points

The constitutive norms are the basis for the decisions of most stakeholders, who can interact and agree on the overall health decision-making process, as they are state and federal health authorities. Table 3 shows the characteristics of the actors according to the interactive decision-making process, and excerpts of interviews are presented as evidence. The Ministries identified themselves as responsible for the formulation of policies for the protection and care of people with MD and considered them within the vulnerable population group. While the directors, mentioned their actions in a more local scenario of concern, participating in internal regulations such as protocols of specific attention to COVID-19 in specialized mental health institutions:

“*The regulations emanate mainly from the Mexican Constitution. Hence derived the Constitution of the State of..., the Federal Health Law, the State Health Law and the Health Sector Plan which is where we take all the elements... to be able to implement the different policies... of this secretariat.” Actor-CS1ss*

“*... a protocol for COVID, we were the first to do it. And yes, in that sense we are a bit of a reference. Those are the public policies to face Covid, and well, there is a national policy of restructuring the National Mental Health Program that is to invest more in primary healthcare, make the second level and we are the third level of care.” Actor-FE1ss*

Decision-makers adopted different measures using power resources through transactions of different natures and scopes, targeting different population groups (see Table 3).

“*... concerning mental disorders, although we have taken action, we have fallen short because of the confinement in which the population has been. We try to push some programs through health services... they have a specific area that has to do with mental health to support them through video claims...” Actor-CS1ss*

“*... the Health Caravans, which are mobile medical units that go to those rural communities which do not have quick access to a Health Center, and through them all those vulnerable, disabled people and those you mentioned are promoted and monitored.” Actor-OE1ss*

About funding, seven of the actors expressed having the power to decide the use and allocation of resources (see Table 3).

“*... here we also had to redistribute the budgets of all the items that arrive.., make a redistribution of all those funds, of the economics, to allocate them to priority actions that were going to have to do with the care of COVID and of course from the epidemiology area of each of the health regions of the hospitals' local care protocols were established to be able to define the treatment strategy...” Actor-OE3ss*

Regarding the organization of healthcare services during the pandemic, to support the guidelines for action in mental health issued by the Federal Ministry of Health, most of the actors involved reported actions under a proactive and interactive behavior but of local scope:

“*In the hospital we made a protocol for the management of this pandemic, among the things we required was the protective equipment for the staff, modify any of the facilities of the hospital; from toilets at the entrance to inside hospital areas, such as two special offices for potential COVID patients; we decreased the admission to 30%, and the flow of outpatient patients and made calls, that is, consultations by video call,... basically it is the follow-up of patients through electronic methods to prevent them from entering, respiratory and psychiatric triage, our two lines of attention to the public and COVID people...” Actor-FE1ss*

Regarding the capacity for education and intersectoral action, some actors described high levels of participation of various sectors of activity in mental health policy formulation:

“*... the National Committee for Health Safety, is a collegiate body that was established in 2002 and whose... attributions or functions are precisely to coordinate the preparation and response to phenomena... that can produce threats to health security, I have coordinated the different working groups that depend directly on me which are eight general directors... who work in coordination with us the National Center for Blood Transfusion and... Psychiatric Care Services,... and the... National Commission Against Addictions.” Actor-FE2ss*

“*... they gave us the task of coordinating the other institutions of the state: the IMSS, the ISSSTE, the private hospitals, the National Defense Secretariat so that through... we concentrate this information and make a report; from the clinical area we pass it to epidemiology of the Ministry of Health and... all this is the final report that is taken to the cabinet and to the office of the secretary or the governor where the decisions of public policies are made.” Actor-OE3ss*

In terms of research, most actors exercised their power in capacities to control the development and implementation of health policies in general:

“*We rely a lot on expert people like people from the National Institute of Public Health, people from UNAM who are developing models at the national level, we are making measurements daily to see our trends in hospital occupancy, our trends of increase in cases, lethality, mortality.” Actor-OE3ss*

However, only one actor mentioned the ability to generate and disseminate mental health information:

“*... we are, as a psychiatric hospital, the largest in the country and in that sense our voice is heard; we are... reference for the other hospitals, and... for... vulnerable groups, we are always in contact, they come to us here... Indigenous... beaten women, the people who are,... in street situation and patients living with... HIV, people living with these psychosocial conditions.” Actor-FE1ss*

In regard to transparency and accountability, all actors expressed transparency mechanisms:

“*As for the Secretariat, there are messages from the governor, from the Ministry of Health in different media, including social networks, and already in the hospital there are many posters and this kind of thing.... I don't know at the level of the Secretary of Health; I know they have a very strict level of control of resources and transparency, but I don't know if they implemented new strategies.” Actor-NOE8ss*

**Figure 2:**
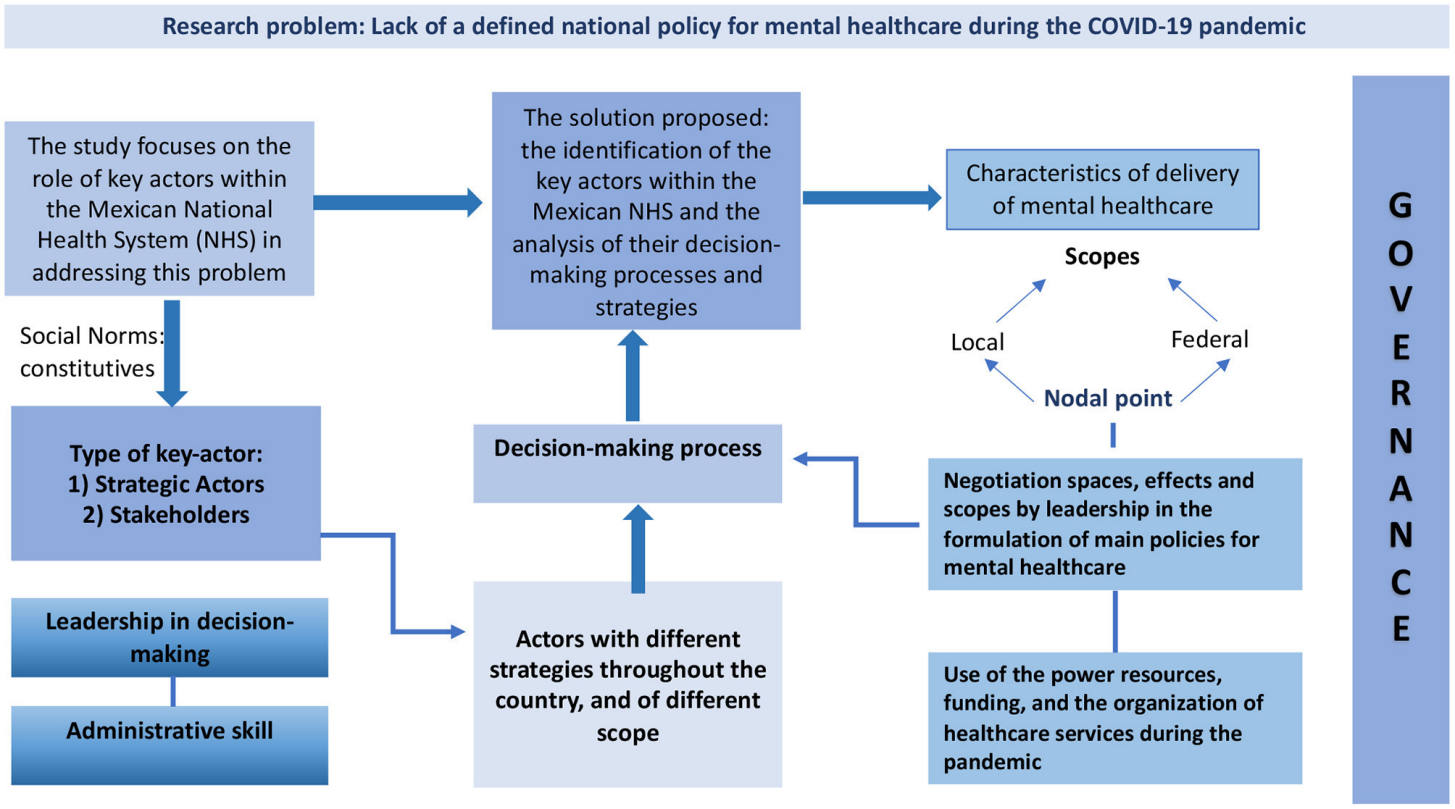
Theoretical contribution of the research: examination of the governance approach to mental healthcare during a pandemic, México, 2020.

Figure 2 summarizes the findings, explaining the research problem, the solution, and the theoretical contribution of the present study.

## 5. Discussion

The results of this study show the heterogeneity in decision-making for the protection and care of people with mental disorders in the context of a health emergency such as the current COVID-19 pandemic. A legislative framework lacking a General Mental Health Law at the national level in Mexico, makes it unclear what actions should be taken to guarantee healthcare access for people with mental disorders, as dictated by the Magna Carta [Const.], 2021 (38). The World Health Organization, in its reports called “Mental Health Atlas”, has stated on more than one occasion the need to address the problem of mental health in a comprehensive manner (with public policies, legislation and financing). In these reports, the region made up of the United States and Canada leads the way with improved scores for the indicators regarding the enactment and updating of laws and the implementation of public policy on mental health (39).

The fact that the Mexican NHS is fragmented in terms of the structure and function of healthcare services, further intensified the problem and limited the responsiveness of decision-makers to decentralize guidelines to state and local contexts. This resulted in the implementation of diverse strategies across the country, and of varying scope, depending mainly on the resources that key actors put at stake, but generally showing a local scope of their actions with little connectivity between the different NHS settings.

Concerning mental healthcare strategies, as reported in the literature, they were more focused on clinical care in the context of the pandemic, i.e., for the population presenting symptoms associated with lockdown and social distancing (40–42), while healthcare services for people with specific MD decreased during the pandemic. Healthcare institutions found it necessary to reduce inpatient and outpatient care processes to implement processes for the detection, monitoring and surveillance of COVID-19 cases. The decline in care for people with MD conflicts with strategies recommended in the scientific literature (43, 44). Leaving these people in a scenario of increased vulnerability, as they may develop greater disease awareness and greater exposure to infectious diseases, such as COVID-19 (45), and less access to available healthcare services, may intensify pre-existing inequality (6, 9, 43).

Thus, the COVID-19 health crisis has shown that the health system in Mexico, as in most countries, was not sufficiently prepared to respond in a reliable and timely manner to the problem (46), and in the case of mental healthcare for people with MD, the problem was even more evident because the access to services became more difficult, and the alternative use of digital/telephonic services was not sufficient (9, 43, 47). Furthermore, those most in need of mental healthcare are those whose livelihoods have been made even more precarious because of social disparities, in turn, few of them will seek help because their basic needs are not met by the mental healthcare systems (9). In contrast, a study in Brazil reported that, the reorganization of healthcare services integrating mental care is necessary to provide care access and continuity of care for people with MD (42).

The fragility of governance in decision-making for the protection and care of people with MD in health crisis scenarios, is partly due to the absence of a specific legislative framework. Indeed, although mental healthcare is included in the Magna Carta, it does not make a clear reference to this type of problem. Mental healthcare service mentions that the Law will define the bases and modalities for access to health services [GLH] (48), which highlights the urgent need for such a law (49).

In the current scenario and concerning the care of people with MD, we found no evidence of any call for decision-makers to interact in decision-making spaces, much less those responsible for mental healthcare. Another aspect to consider in this pandemic context is the fact that to provide continuity of clinical care, the healthcare system could resort to telematic services, but the bill to provide legal protection to health professionals and users of these services has been canceled in Mexico (50). This absence of a legal regulatory framework can also be observed in the countries of the European Union (51–53).

On the other hand, despite the decentralization of healthcare services in Mexico, unilateral and centralized decision-making enforced during the pandemic, diminished proactive interest in participating in the actions described in the policies (54). While the NHS follows—according to key informant actors—official constitutive-regulatory norms, our analyses show leadership capacity as an essential characteristic of the decision-maker to undertake the formulated actions and a key element to strengthening healthcare system governance (55–57). Further, it is mandatory that all levels of government invest in mental healthcare, not only to offset the pandemic but also to support thriving in the future for people with MD (9, 58–60).

Strengthening governance in healthcare systems involves knowing, convening, and agreeing to make proactive decisions in the formulation of comprehensive and equitable policies, including care for the most vulnerable groups in society, such as those with MD (61). A process that requires leading decision makers with strong social values (62) to design suitable strategies to overcome the barriers to access to mental healthcare services (63). It is evident that, the sectoral and multi-scalar healthcare structure of the NHS in Mexico gives greater complexity to the analysis of the decision-making process in the field of mental healthcare, due to the interaction of multiple actors with differing interests, roles and levels of responsibility. To adapt the healthcare services to the care needs of the population in the absence of a national policy of mental healthcare, decision-makers must create an adaptative team management, with cohesion, collaboration, leadership, guidance and direction from management in providing sustained, efficient, and equitable delivery of mental healthcare for people with MD during a sanitary emergency such as the COVID-19 pandemic (41).

In summary, the study shows that the lack of a national mental health law in Mexico and the fragmented structure of the healthcare system have made it difficult for decision-makers to provide adequate care for people with mental disorders during the COVID-19 pandemic. The focus of mental healthcare strategies has been primarily on addressing symptoms associated with lockdown and social distancing, rather than on providing care for people with specific mental disorders. This has led to a decline in access to care for people with mental disorders, which has made them more vulnerable to the pandemic. The study also highlights the need for a specific legislative framework to guide decision-making in the protection and care of people with mental disorders during health crises. Additionally, it emphasizes the importance of leadership capacity and proactive decision-making in strengthening governance in the healthcare system and investing in mental healthcare to support the wellbeing of people with mental disorders in the future.

The limitations of this study include: (1) a limited sample size of key-actors, which could restrict the generalizability of the findings to the larger population of Mexico. (2) The fact that it was based on self-reported data, which could be subject to bias or inaccuracies in the recall. (3) The study only focuses on one specific aspect of governance, which may not fully capture the complexity and nuances of decision-making in the health system. (4) It is possible that the study only considered the perspectives of certain groups of actors and not others, which could limit the scope of the findings. (5) It is a qualitative study, which makes it difficult to generalize the findings. (6) The study only analyzes the situation of a particular health emergency, which makes it difficult to generalize the findings to other types of emergencies. Despite these limitations, the results of the study can be considered reliable in terms of reflecting the way decisions are typically made in the health system in Mexico.

## Data availability statement

The datasets presented in this article are not readily available due to the informed consent with the participants, in which it was agreed that their data would not be shared apart from the research team.

## Ethics statement

This study was approved by the Committee for Ethics in Investigation of the National Institute of Psychiatry (Mexico), 17 June 2020. Protocol code CEI/C/017/2020. All participants provided valid informed consent to get involved in the study, verbally, which was recorded at the time of the interviews.

## Author contributions

LDC contributed to the design, data analysis, and interpretation and writing of the first and subsequent drafts of the paper. JCSH contributed to the data analysis and interpretation and writing of the first and subsequent drafts of the paper. OOGR, EON, and MSSD contributed to the interpretation and writing of subsequent drafts of the paper. All authors contributed to the article and approved the submitted version.
